# Socio-demographic and lifestyle factors associated with overweight in a representative sample of 11-15 year olds in France: Results from the WHO-Collaborative Health Behaviour in School-aged Children (HBSC) cross-sectional study

**DOI:** 10.1186/1471-2458-11-442

**Published:** 2011-06-07

**Authors:** Marie Dupuy, Emmanuelle Godeau, Céline Vignes, Namanjeet Ahluwalia

**Affiliations:** 1 INSERM U1027, Epidémiologie et Analyses en Santé Publique, Toulouse, France; 2 Université Paul Sabatier Toulouse III, Toulouse, France; 3 Service Médical du Rectorat de Toulouse, Toulouse, France; 4 Faculty of Medicine, University of Paris 13, INSERM U557, Bobigny, France

**Keywords:** overweight, adolescence, socio-economic status, diet, physical activity and sedentary behavior, smoking, alcohol consumption

## Abstract

**Background:**

The prevalence of overweight in children and adolescents is high and overweight is associated with poor health outcomes over short- and long-term. Lifestyle factors can interact to influence overweight. Comprehensive studies linking overweight concomitantly with several demographic and potentially-modifiable lifestyle factors and health-risk behaviours are limited in adolescents - an age-group characterized by changes in lifestyle behaviours and high prevalence of overweight. Thus, the objective of the current study was to examine the association of overweight with several socio-demographic and lifestyle variables simultaneously in a representative sample of adolescents.

**Methods:**

A nationally representative sample of 11-15 year-olds (n = 7154) in France participated as part of the WHO-Collaborative Health Behaviour in School-aged Children (HBSC) study. Students reported data on their age, height, weight, socio-demographic variables, lifestyle factors including nutrition practices, physical activity at two levels of intensity (moderate and vigorous), sedentary behaviours, as well as smoking and alcohol consumption patterns using standardized HBSC protocols. Overweight (including obesity) was defined using the IOTF reference. The multivariate association of overweight with several socio-demographic and lifestyle factors was examined with logistic regression models.

**Results:**

The adjusted odds ratios for the association with overweight were: 1.80 (95% CI: 1.37-2.36) for low family affluence; 0.73 (0.60-0.88) for eating breakfast daily; 0.69 (0.56-0.84) for moderate to vigorous physical activity (MVPA); and 0.71 (0.59-0.86) for vigorous physical activity (VPA). Significant interactions between age and gender as well as television (TV) viewing and gender were noted: for boys, overweight was not associated with age or TV viewing; in contrast, for girls overweight correlated negatively with age and positively with TV viewing. Fruit and vegetable intake, computer and video-games use, smoking and alcohol consumption were not associated with overweight.

**Conclusions:**

In multivariate model, family affluence, breakfast consumption and moderate to vigorous as well as vigorous physical activity were negatively associated with overweight. These findings extend previous research to a setting where multiple risk and protective factors were simultaneously examined and highlight the importance of multi-faceted approaches promoting physical activity and healthy food choices such as breakfast consumption for overweight prevention in adolescents.

## Background

Overweight remains a public health problem in children and adolescents worldwide [1,2]. In a nationally representative sample of children in France, the prevalence of overweight among 11-14 year olds was 15.2% in 2006/07 and did not change versus preceding survey in 1998/99 [3].

Overweight in childhood and adolescence has been associated with several short and long-term adverse health consequences [1,4]. These include increased risk of cardiovascular disease and related metabolic anomalies such as dyslipidemia and impaired glucose tolerance and increased likelihood of being obese in adulthood, other conditions such as sleep apnea and orthopedic problems, as well as several psychological and social repercussions [1,4]. Furthermore, the care of overweight individuals is associated with increased health care costs to the society [1].

The etiology of overweight is complex and involves interplay of genetic predisposition with environmental factors including socio-demographic variables as well as potentially modifiable lifestyle factors. The latter can include nutrition practices, physical activity and sedentary behaviours, as well as health-risk behaviours such as smoking and alcohol consumption. Most of the previous literature in children has examined the link of overweight with these factors either singly [2,5-7]; or in combination with certain lifestyle or health-risk factors [8-13] in a wide age range of participants rather than in adolescents per se [12,14]. Such studies generally report an inverse association between socio-economic status (SES) and overweight in developed countries [3,15]. In addition, skipping breakfast has generally been linked to an increased probability of being overweight [2,14,16,17]. Concerning the association of fruit and vegetable intake with overweight, however, the evidence is less consistent [8,9,14,18]. Although majority of studies support that childhood physical activity is negatively related to overweight and measures of body fat [2,8,10,19,20] not all studies examined different levels of physical activity in relation to its intensity (moderate/intense). Studies examining sedentary behaviours have considered television (TV) viewing, computer and video game use, separately or in combination, in relation to overweight [9,10,14,17,21-23]. A positive association between overweight and TV viewing, and less consistent associations of overweight with computer use and video games have been reported [8,19,24]. In addition, few studies have examined the association of health-risk behaviours such as smoking and alcohol consumption practices with overweight in adolescents [5,6,11,17]; most reported a positive association for overweight and smoking [6,11] as well as for overweight and alcohol consumption [5,6,17] but others did not find any relation for BMI and smoking or alcohol [25]. It is important to examine alcohol and smoking practices in relation to overweight because these practices could impact energy metabolism and thereby affect overweight [26,27].

Because lifestyle factors can interact with each other in a synergistic or antagonistic manner to influence overweight prevalence, it is particularly important to examine the association of overweight with several factors considered simultaneously including socio-demographic and various potentially modifiable lifestyle factors (eating habits, physical activity examined at different levels of intensity, and sedentary behaviours), and health-risk behaviours. Although, some studies have examined the contribution of certain factors concomitantly in multivariate models in pre-teens and adolescents [8-10,17], only a few have included socio-demographic factors, diet, physical activity and health risk behaviours simultaneously in multivariate models [14,17]. These studies show positive associations of overweight with TV viewing [14], alcohol intake [17], and smoking [14]; as well as negative associations of overweight with breakfast [14,17], and fruit and vegetable consumption [14]. Furthermore, most studies have generally included a wide age range of participants [3,12,14]; thus these results may not be specific to adolescents who are likely to have behaviours different from children and adults. Given the challenges in treating overweight and obesity, prevention of excessive weight gain is considered the only feasible solution [1]. Thus, the identification of factors that are associated with overweight in a thorough multivariate context in adolescents is important, because it could help prioritize areas for developing educational messages and preventive programs targeting such factors, to potentially reduce overweight and associated health concerns in young persons.

The objective of the current study was to identify socio-demographic and lifestyle factors associated with overweight in a nationally representative sample of 11-, 13-, and 15-year old boys and girls in France participating in the WHO-collaborative Health Behaviour in School-Aged Children (HBSC) survey in 2006.

## Methods

Data for present analyses were collected using standardized research protocols [28] for the 2006 WHO-collaborative HBSC study in France, with the aim of gaining insights into adolescents' health and health behaviours. A nationally representative sample of 11-, 13-, and 15-year olds was selected with school class as the sampling unit.

Data were collected in 715 classes (701 schools); 7154 school-aged children (3558 boys, 3596 girls) participated in the survey. Questionnaires were administered in class and all data were self-declared. Participation was voluntary, and anonymity and confidentiality were ensured. Parental informed consent and child assent were obtained for all participants following protocol approved by the French National Commission of Information and Liberty ("Commission Nationale de l'Informatique et des Libertés"). For the present analyses children who did not report their age, weight or height were excluded (n = 550) leaving 6604 (3284 boys and 3320 girls) pupils (92.3%).

*Body Mass Index (BMI) *(kg/m^2^) was calculated using self-reported weight and height. Overweight was defined using age- and gender-specific cut-offs recommended by the International Obesity Task Force [29] corresponding to adult BMI ≥25 kg/m^2^.

*Family affluence *was determined with the Family Affluence Scale as a score of four items [30]: Does your family own a car, van or truck? (0-2 points). Do you have your own bedroom for yourself? (0-1 points). During the past twelve months, how many times did you travel away on holiday (vacation) with your family? (0-2 points); and how many computers does your family own? (0-2 points). Responses were recoded: "low", "medium" and "high".

### Lifestyle variables

Usual eating habits were assessed by asking participants how many times a week they eat fruit and vegetables. The possible responses were: "never", "less than once a week", "about once a week", "two to four days a week", "five to six days a week", "once a day, every day", "every day, more than once". For each of these variables, responses were dichotomised: "less than daily" and "daily" [31].

To assess regular breakfast consumption, students were asked to estimate how many days during the week they had breakfast (i.e. having more than a glass of milk or fruit juice). Responses were recoded: "less than daily" versus "daily" [31].

Moderate to vigorous physical activity (MVPA) was assessed by asking: "On how many days in the past week were you physically active for 60 minutes or more". Physical activity was defined as "any activity that increases your heart rate and makes you get out of breath some of the time" with examples of such activities. Response categories were: "0 days", "1", "2", etc up to "7 days", recoded as < or ≥ 5 days per week [32]. Vigorous physical activity (VPA) was assessed by asking: "Outside school hours, how many hours a week do you usually practise sports in your free time so much that you get out of breath or sweat?" These questions have been shown previously to be reliable and valid [33]. Response categories were none, about 30 minutes, and 1, 2-3, 4-6, ≥7 hours; recoded into < or ≥ 2 hours per week [34].

Three items assessed sedentary screen-based activities during the week: 1) "About how many hours a day do you usually watch television (including DVDs and videos) in your free time?" 2) "About how many hours a day do you usually play games on a computer or games console (Playstation, Xbox, GameCube etc.) in your free time?" and 3) "About how many hours a day do you usually use a computer for chatting on-line, internet, emailing, homework etc. in your free time?" All three items had nine possible responses: "none at all", "about 30 min, 1 hour, 2 hour, up to ≥7 hour/day". Responses were recoded into ≤ versus > 2 hours per day [35].

To determine alcohol consumption level students were asked to report how many times they consumed alcoholic beverages. The responses were never, rarely, monthly, weekly or daily. The responses were recoded into weekly alcohol consumption (yes or no) [34]. In post-hoc analyses, we also considered alcohol consumption using definitions employed by others investigators [5,6,17]. We recoded the data to ascertain whether the adolescents reported consuming alcohol (yes/no). In addition, we also examined the number of times they had been drunk (obtained from the question "how often have you been drunk?").

Smoking status was defined on the basis of the question: How often do you smoke tobacco? Possible responses were: "every day", "at least once a week, but not every day", "less than once a week" or "never", recoded into: non-smokers; smoker, not daily; and smoker, daily [36].

### Statistical Analyses

Statistical analyses were performed using STATA 9.2 (College Station, Texas, USA). The svy, vec (linearized) command in STATA were used to define school class as the primary sampling unit in order to account for intra-class correlation. Univariate associations and corresponding odds ratios (OR) and 95% confidence interval (CI) between overweight status and socio-demographic and lifestyle variables concerning eating patterns, physical activity and sedentary behaviours, as well as health-risk behaviours were obtained with chi-square analysis. All variables were entered simultaneously into a multivariate logistic regression model. Interactions between variables were tested and, when significant, included in the regression model. *P *< 0.05 was considered significant.

## Results

The prevalence of overweight was 10.4%: overweight was more common in boys (11.8%) than in girls (9.0%) (*P *= 0.0002). Statistically significant associations between gender and socio-demographic and lifestyle factors were noted with the exception of age, smoking and computer use (data not shown). Boys reported significantly more often having high family affluence than girls (52.5 vs 48.4%; *P*< 0.01). Weekly alcohol consumption was more frequent in boys (14.7%) than girls (8.2%) (*P*< 0.01). Daily breakfast consumption was more common in boys (63.3%) as compared to girls (56.6%) (*P*< 0.01); in contrast, boys reported significantly less often than girls to consume daily fruit (29.2 vs 33.4%) and vegetables (39.5 vs 45.9%). Using video games (> 2 h/d) and TV viewing (> 2 h/d) was more frequent in boys than girls (31.2 vs 10.5%; *P*< 0.01 and 53.2 vs 48.3%; *P*< 0.01, respectively). Boys declared more frequently than girls to engage in MVPA (≥ 60 min ≥5 days per week) (42.5 vs 25.1%; *P*< 0.01) and VPA (≥ 2 h per week) (66.0 vs 43.2%; *P*< 0.01).

Table 1 shows the univariate associations of overweight with socio-demographic and life-style factors and health risk-behaviours examined in the study. Family affluence, daily breakfast, and physical activity at both levels of intensities were significantly and negatively associated with overweight; while TV viewing was positively associated with overweight. When examined separately by gender, most of these associations were similar between boys and girls. However, age was negatively associated with overweight in girls only, and smoking was positively correlated with overweight in boys only.

**Table 1:** Association of overweight with socio-demographic and lifestyle factors^a^

	ALL			BOYS			GIRLS		
	**Non-overweight** **n (%)**	**Over-weight** **n (%)**	**OR (95% CI)**	**Non-overweight (%)**	**Over-weight** **n (%)**	**OR (95% CI)**	**Non-overweight (%)**	**Over-weight** **n (%)**	**OR (95% CI)**

Age	*P = 0.5232*			*P = 0.0516*			*P = 0.0206*		
11 years	1990 (89.5)	234 (10.5)	1	1016 (89.9)	114 (10.1)	1	974 (89.0)	120 (11.0)	1
13 years	2031 (90.2)	220 (9.8)	0.92 (0.74-1.14)	942 (88.3)	125 (11.7)	1.18 (0.87-1.60)	1089 (92.0)	95 (8.0)	0.71 (0.53-0.94)
15 years	1897 (89.1)	232 (10.9)	1.04 (0.85-1.27)	938 (86.3)	149 (13.7)	1.42 (1.09-1.84)	959 (92.0)	83 (8.0)	0.70 (0.52-0.95)
Family affluence	*P < 0.0001*			*P = 0.0036*			*P < 0.0001*		
High	2956 (91.4)	277 (8.6)	1	1490 (89.8)	170 (10.2)	1	1466 (93.2)	107 (6.8)	1
Medium	2166 (88.9)	269 (11.1)	1.33 (1.12-1.57)	1005 (86.9)	151 (13.1)	1.32 (1.04-1.66)	1161 (90.8)	118 (9.2)	1.39 (1.07-1.81)
Low	617 (83.5)	122 (16.5)	2.11 (1.66-2.68)	289 (84.0)	55 (16.0)	1.67 (1.19-2.34)	328 (83.0)	67 (17.0)	2.80 (2.01-3.89)
Smoking	*P = 0.4455*			*P = 0.0384*			*P = 0.4526*		
No	5175 (89.8)	586 (10.2)	1	2546 (88.6)	326 (11.4)	1	2629 (91.0)	260 (9.0)	1
Yes, but not daily	330 (88.2)	44 (11.8)	1.18 (0.84-1.65)	155 (86.1)	25 (13.9)	1.26 (0.80-1.98)	175 (90.2)	19 (9.8)	1.10 (0.67-1.79)
Yes, daily	310 (88.3)	41 (11.7)	1.17 (0.83-1.64)	136 (82.4)	29 (17.6)	1.67 (1.11-2.51)	174 (93.5)	12 (6.5)	0.70 (0.38-1.28)
Consuming alcohol weekly	*P = 0.1101*			*P = 0.3484*			*P = 0.5029*		
No	5239 (89.9)	590 (10.1)	1	2466 (88.4)	324 (11.6)	1	2773 (91.2)	266 (8.8)	1
Yes	663 (88.0)	90 (12.0)	1.21 (0.96-1.52)	418 (86.9)	63 (13.1)	1.15 (0.86-1.53)	245 (90.1)	27 (9.9)	1.15 (0.76-1.73)
Eating fruit daily	*P = 0.5701*			*P = 0.5285*			*P = 0.9415*		
No	4024 (89.5)	471 (10.5)	1	2022 (87.9)	277 (12.1)	1	2002 (91.2)	194 (8.8)	1
Yes	1844 (90.0)	205 (10.0)	0.95 (0.79-1.13)	842 (88.7)	107 (11.3)	0.93 (0.73-1.17)	1002 (91.1)	98 (8.9)	1.01 (0.79-1.29)
Eating vegetables daily	*P = 0.2157*			*P = 0.3607*			*P = 0.6648*		
No	3344 (89.2)	403 (10.8)	1	1725 (87.7)	241 (12.3)	1	1619 (90.9)	162 (9.1)	1
Yes	2518 (90.2)	275 (9.8)	0.91 (0.78-1.06)	1138 (88.8)	144 (11.2)	0.91 (0.73-1.12)	1380 (91.3)	131 (8.7)	0.95 (0.75-1.20)
Eating breakfast daily	*P = 0.0011*			*P = 0.0008*			*P = 0.0840*		
No	2260 (88.0)	308 (12.0)	1	996 (85.6)	168 (14.4)	1	1264 (90.0)	140 (10.0)	1
Yes	3482 (90.7)	356 (9.3)	0.75 (0.63-0.89)	1802 (89.7)	207 (10.3)	0.68 (0.54-0.85)	1680 (91.9)	149 (8.1)	0.80 (0.62-1.03)
MVPA ≥ 1 hour for ≥ 5 days per week	*P < 0.0001*			*P < 0.0001*			*P = 0.0020*		
No	3806 (88.4)	498 (11.6)	1	1599 (86.2)	256 (13.8)	1	2207 (90.1)	242 (9.9)	1
Yes	2018 (92.0)	176 (8.0)	0.67 (0.56-0.80)	1248 (90.8)	126 (9.2)	0.63 (0.51-0.78)	770 (93.9)	50 (6.1)	0.59 (0.42-0.83)
VPA ≥ 2 hours per week	*P < 0.0001*			*P < 0.0001*			*P = 0.0001*		
No	2600 (87.5)	370 (12.5)	1	930 (84.6)	169 (15.4)	1	1670 (89.3)	201 (10.7)	1
Yes	3249 (91.3)	308 (8.7)	0.67 (0.57-0.78)	1920 (90.1)	212 (9.9)	0.61 (0.49-0.75)	1329 (93.3)	96 (6.7)	0.60 (0.46-0.77)
TV viewing > 2 hours per day	*P < 0.0001*			*P = 0.1024*			*P < 0.0001*		
No	2887 (91.6)	266 (8.4)	1	1318 (89.2)	160 (10.8)	1	1569 (93.7)	106 (6.3)	1
Yes	2848 (87.8)	397 (12.2)	1.51 (1.28-1.79)	1466 (87.3)	213 (12.7)	1.20 (0.96-1.49)	1382 (88.2)	184 (11.8)	1.97 (1.51-2.57)
Using video games > 2 hours per day	*P = 0.1437*			*P = 0.4494*			*P = 0.8691*		
No	4609 (90.0)	514 (10.0)	1	1942 (88.6)	250 (11.4)	1	2667 (91.0)	264 (9.0)	1
Yes	1184 (88.6)	153 (11.4)	1.16 (0.95-1.41)	871 (87.6)	123 (12.4)	1.10 (0.86-1.39)	313 (91.3)	30 (8.7)	0.97 (0.66-1.42)
Using computer > 2 hours per day	*P = 0.1916*			*P = 0.8723*			*P = 0.0867*		
No	4314 (89.4)	511 (10.6)	1	2101 (88.1)	283 (11.9)	1	2213 (90.7)	228 (9.3)	1
Yes	1429 (90.5)	150 (9.5)	0.89 (0.74-1.06)	681 (88.3)	90 (11.7)	0.98 (0.78-1.24)	748 (92.6)	60 (7.4)	0.78 (0.58-1.04)

^a^: *P *obtained with chi-square test; MVPA: moderate to vigorous physical activity, VPA: vigorous physical activity, TV: television

Table 2 shows the findings from multivariate regression model including all the factors examined and the significant interaction terms (age*gender and TV viewing*gender). Family affluence, daily breakfast, and physical activity at both levels of intensities were significantly and negatively associated with overweight in adolescents (boys and girls combined). A significant interaction was noted for gender with age and TV viewing. Specifically, for girls age was negatively associated with overweight while no association was found for age and overweight in boys. With respect to TV viewing, for girls a positive association was observed with overweight whereas for boys no association was noted.

**Table 2:** Multivariate association of overweight with socio-demographic and lifestyle factors

	OR	95% CI	*P*
Family affluence			*0.0001*
High	1		
Medium	1.24	1.03-1.51	0.024
Low	1.80	1.37-2.36	< 0.001
Smoking			*0.7468*
No	1		
Yes, but not daily	1.14	0.78-1.65	0.498
Yes, daily	1.11	0.73-1.69	0.620
Consuming alcohol weekly			
No	1		
Yes	1.03	0.77-1.38	0.823
Eating fruit daily			
No	1		
Yes	1.10	0.90-1.36	0.352
Eating vegetables daily			
No	1		
Yes	1.01	0.84-1.22	0.886
Eating breakfast daily			
No	1		
Yes	0.73	0.60-0.88	0.001
MVPA ≥ 1 hour for ≥ 5 days per week			
No	1		
Yes	0.69	0.56-0.84	< 0.001
VPA ≥ 2 hours per week			
No			
Yes	0.71	0.59-0.86	< 0.001
Using video games > 2 hours per day			
No	1		
Yes	0.97	0.76-1.23	0.795
Using computer > 2 hours per day			
No	1		
Yes	0.89	0.72-1.10	0.276
Interaction age*gender			
11 years*boys	1		
13 years*boys	1.14	0.83-1.58	0.415
15 years*boys	1.27	0.93-1.74	0.130
11 years*girls	1		
13 years*girls	0.68	0.49-0.92	0.014
15 years*girls	0.55	0.39-0.78	0.001
Interaction TV viewing *gender			
TV viewing (≤ 2 hours per day)*boys	1		
TV viewing (> 2 hours per day)*boys	1.21	0.94-1.54	0.133
TV viewing (≤ 2 hours per day)*girls	1		
TV viewing (> 2 hours per day)*girls	1.93	1.42-2.62	< 0.001

MVPA: moderate to vigorous physical activity, VPA: vigorous physical activity, TV: television

## Discussion

Previous studies have reported on the multivariate association of overweight with certain modifiable factors [8-13] but only a few studies examined several socio-demographic and lifestyle factors including health-risk behaviours concomitantly [14,17]. This study is the first to examine the association of overweight with several socio-demographic and potentially modifiable lifestyle factors simultaneously, in a representative sample of 11, 13- and 15-year old boys and girls in France. These factors included not only traditional socio-demographic variables and lifestyle factors such as eating habits, physical activity (examined at two levels of intensity), and several sedentary behaviours, but also health-risk behaviours namely alcohol consumption and smoking. Our finding of significant association between overweight and breakfast as well as overweight and physical activity in adolescents fits well with the literature in general and in studies examining multiple factors simultaneously [14,17]. Previous studies that employed a similar approach as the current study, considering several lifestyle factors that can affect overweight simultaneously in multivariate models, had adjusted for gender in their analysis [14,17]. In the current study we did not adjust for gender because we found certain significant interactions (gender*age; and gender*TV viewing). Specifically, in girls a negative association between overweight and age was seen in the current study as been reported previously in an analysis in several countries [2]. In addition, we observed a positive association between overweight and TV viewing for girls only, as has been shown in a few studies [12,21,37]. Taken together these findings suggest that the interaction of gender with lifestyle variables that could affect overweight should be examined further in future studies to develop gender-specific prevention messages as needed [38,39]. Moreover, in the current study both moderate- and vigorous physical activity were significantly associated with reduced odds of overweight in both genders. Few studies have examined the association of overweight with physical activity at different intensities [10,20]; these studies found that the negative correlation between physical activity and overweight or body fat was stronger for VPA than MVPA. In addition, smoking and alcohol consumption were not associated with overweight in this population, although findings of a positive [6,11] or no association [25] have also been reported previously.

Among the socio-demographic variables examined in the study, a consistent negative association between family affluence and overweight was noted. This is congruent with several, but not all, previous reports in developed countries (including USA, Canada, and North and Western European countries) particularly among Caucasian populations [3,15,22,40,41]. In the current study, we observed a negative association between age and overweight in girls only. The literature on the association of overweight with age during adolescence is mixed [2]; reporting no association [10,12], positive [13] or negative association [22].

We examined three domains of lifestyle factors that could be related to overweight and obesity, namely breakfast consumption, fruit and vegetable intake patterns; physical activity and sedentary behaviours; and health-risk behaviours that could impact energy metabolism such as alcohol consumption and smoking habits. Interestingly the only dietary variable that remained strongly associated with overweight in multivariate analysis was breakfast skipping. There is a vast literature suggesting that regular breakfast consumption is negatively associated with overweight or BMI [2,11,14,16,17]; this finding is usually reported for both genders as was noted in the current study.

No associations between fruit and vegetable consumption and overweight were observed in the current study. This finding is consistent with other reports [2,8] and affirms the conclusion of a recent review [18] that available data do not support a protective effect of fruit and vegetable consumption on the risk of childhood obesity; although contrary findings have also been reported [14]. Most studies reported in the review by Newby [18] have used food frequency questionnaires to assess dietary intakes which may not provide as precise estimates as repeated 24-hour food recall or food records. However, studies relying on latter methods reported mixed findings [18] and use of such methods is unfeasible in large surveys involving representative samples that collect data on multiple lifestyle determinants in children and youth such as the current study.

We examined physical activity during the week in two ways: MVPA concerning regular daily activities including sports at school, and VPA which was defined as participation in sports outside school hours and involved a more intense activity component (being out of breath or breaking into sweat). In the current study both physical activity variables were negatively and significantly associated with overweight in both boys and girls. Most studies have not differentiated the effects of physical activity in terms of its intensity with the exception of a few [10,20,42]. In a relatively small study (n = 878) Patrick et al. [10] found a significant association between overweight and VPA but not with moderate physical activity. Overall, childhood physical activity has usually been negatively related to overweight [2,8,17,43]; however others have found a positive association in boys [44] or in girls only [12]. No correlation between BMI and physical activity has also been reported [45] despite the biologically plausible mechanisms involving increased energy expenditure subsequent to physical activity. Our findings are of public health interest because they indicate that both moderate-to-vigorous and vigorous levels of physical activity can have beneficial effects on overweight in adolescents. This finding is supported by the recent report by Moliner-Urdiales and colleagues [20] indicating that several measures of body fat were associated with moderate and vigorous physical activity. Taken together, these findings suggest that physical activity at moderate or vigorous level should be promoted in young persons to reduce overweight prevalence.

Sedentary behaviours can contribute to overweight due to reduced energy expenditure as well as associated snacking [7]. In the current study no associations were noted between overweight and video games or computer use in boys or girls. Few studies have investigated the association of these sedentary behaviours with overweight in a multivariate context, and their findings generally support a lack of an association with overweight [2,8,9,24]. However, a positive association between overweight and video game use has also been reported [43]. In addition, BMI and overweight have been shown to be associated with computer use in teenage girls only [7,21]. Stronger positive associations have usually been reported with TV viewing in both genders in several studies [7,8,24,43]. However, in the current study, TV viewing was positively associated with overweight in girls only. This is consistent with other reports [12,21,37] even though a few studies have shown a positive association in boys only [9,46]. It is likely that sedentary practices are culture-specific and influenced by gender within given cultural contexts.

Combined effects of physical activity and sedentary behaviours (TV viewing; TV and video games considered together) have also been investigated in a few studies because of their potential interaction [23,42,44]. Some evidence of an interaction between TV viewing and physical activity has been noted previously particularly in girls [42]. In the study by Eisenmann and colleagues [42], engaging in physical activity compensated the adverse effects of moderate to high TV viewing (at least 2 hours per day) on overweight prevalence, particularly in girls. Similar findings were noted by Laurson and colleagues [23] considering time spent on TV viewing and video games: in girls who spent > 2 hours on TV and video games, the risk of overweight was greater in those who did not meet the physical activity recommendation. Given that many adolescents may practise these behaviours simultaneously, we examined the interactive effects of physical activity variables with TV viewing during the week as well as during week days only. There was no significant interaction between TV viewing over 7 days and MVPA (or VPA). However, for TV viewing during week days only, a significant interaction was noted between TV viewing and MVPA (but not with VPA). For students who engaged in ≥ 60 min of MVPA (≥ 5 days per week) TV viewing was not associated with overweight, i.e. meeting this level of MVPA compensated the negative effects of TV viewing. In contrast, in adolescents who did not reach this MVPA level, TV viewing > 2 hours per week day was significantly associated with increased odds of overweight (OR:1.47 and 95%CI: 1.18 - 1.85). This interesting finding suggests that physical activity is a stronger determinant of overweight than sedentary behaviours in adolescents.

Few studies have examined the association of health-risk factors such as smoking and alcohol consumption with overweight in adolescents using univariate or multivariate approaches [5,6,11,17]. Despite the commonly-held belief that adolescents, particularly girls, use smoking as a means to control body weight [47], no association between smoking status and overweight was noted in the current study in the multivariate models. The findings in the literature on this topic are limited particularly among adolescents and are generally inconsistent ranging from either no association [25], to a negative [48] or positive association [6,11,49]. Interestingly, the positive association between smoking and overweight was noted more often in girls in the latter studies [6,11,49], which was not confirmed by our findings. The current study is an important addition to the existing literature on the relationship of smoking and body weight in adolescents. The finding of a lack of association between smoking and overweight could be of public health importance given that often adolescents may engage in smoking behaviour to control their body weight [47], and this needs to be verified in future studies.

The few European studies that have examined alcohol intake and overweight in pre-teens and adolescents usually report a positive association [5,6,17]. These studies have used differential definitions for alcohol consumption [5,6,17]. In the current study we used weekly consumption of alcoholic beverages (yes/no) and found no association with overweight. In post-hoc analyses we considered alcohol intake as used by other authors [5,6], namely consume alcohol (yes/no) and number of times been drunk, the finding of a null association between alcohol consumption and overweight remained. In the current study, weekly alcohol consumption was reported by 8.2% girls and 14.7% boys; it is likely that the cultural differences in alcohol consumption practices in youth across countries [50] could contribute to differential findings across studies.

It is important to consider certain limitations in the study. The current results concerning overweight and lifestyle factors were based on self-reported data that could be subject to socially desirable reporting bias. However, honest reporting was encouraged by telling students that their responses were anonymous and by ensuring confidentiality. BMI based on self-reported data can underestimate the prevalence of overweight compared to those based on actual height and weight measurements [51]; others have reported high accuracy for classification of youth as obese or non-obese based on self-reported data [52]. BMI based on self-reports has been found to be fairly reliable [52] and suitable for identifying valid relationships in epidemiological studies [52,53]. Associations between weight status and lifestyle factors (e.g. physical activity, TV viewing, breakfast habits) did not differ when based on self-reported versus measured height and weight data [52]. However, whether self-reported data concerning BMI could have induced bias in the study findings can not be ruled out. For instance, the underestimation of BMI based on self-declared data is noted more in girls (versus boys) and more often at older ages [54-56]. Thus the finding of a negative association between overweight and age seen in girls in the current study could have been subjected to reporting error. It could be speculated that the lack of an association between fruit and vegetable intake and overweight could be related to the definition used (daily, or less than daily); however when we considered these variables in the original scale the findings were unchanged. Dietary data in the current study were determined based on the frequency of consumption of food items without any details on the quantity consumed that could have attenuated and potentially masked certain associations. It is likely that the associations found in this observational study based on self-declared data could represent a myriad of other lifestyle variables (e.g. social factors) that were not examined in the study. In addition these findings could be affected due to the relative imprecision of some of the measures used as confounders.

In the current study, 7.7% of the sample had missing values on BMI. A high proportion of missing data on height and weight is usually reported in this age group across countries [8]. To examine the possibility of a selection bias, we compared the "non-responders" (students that did not provide either age, height or weight data to allow the estimation of BMI) with the "responders" (for whom BMI data were available). For most variables (breakfast intake, fruit consumption, TV viewing, computer use, video game use, and smoking) there were no differences among responders and non-responders; however, differences were noted for a few variables (vegetable intake, physical activity and alcohol consumption) which could have affected their associations with overweight in the study. Finally, the cross-sectional nature of this study does not allow making causal inferences.

## Conclusion

In conclusion, the current study involving a large representative sample of 11 to 15 year old students, concerning simultaneous examination of the association of several socio-demographic and lifestyle factors with overweight, provides important findings concerning the role of family affluence, physical activity, TV viewing and breakfast consumption and the need for examining their interactions. Our findings extend previous results on this subject in a setting where multiple risk and protective factors were simultaneously examined among adolescents in France, and highlight the importance of multi-faceted approaches promoting physical activity and healthy nutrition practices such as breakfast consumption for overweight prevention in adolescents. The current study suggests that prevention of overweight in youth may involve certain common messages across genders such as the importance of breakfast consumption and moderate physical activity routinely. Further research on understanding the importance of gender differences in factors that can affect overweight such as TV viewing is needed to guide the formulation of programs and messages for overweight prevention in young persons.

## Competing interests

The authors declare that they have no competing interests.

## Authors' contributions

MD and NA designed the analysis, and wrote the manuscript. MD carried out statistical analysis with the assistance of CV and NA. EG was responsible for study design and coordination. CV participated in data collection and analysis, as well as overall organisation of the study. MD is a doctoral candidate under the supervision of NA and this work represents part of her doctoral research. All authors contributed to manuscript development, and read and approved the final manuscript.

## Pre-publication history

The pre-publication history for this paper can be accessed here:

http://www.biomedcentral.com/1471-2458/11/442/prepub
